# On Chancre

**Published:** 1855-10

**Authors:** 


*On Chancre.* By Professor SIGMUND. From observations conducted on a large scale at the Vienna Hospital, Dr. Sigmund concludes: 1. Chancre can only be treated locally during the first four days, and the farther we recede from this the greater the urgency of the general treatment. 2. The local treatment consists in cauterization, which effectually destroys all the chancrous exudation to the sound tissue. 3. The observation of more than a thousand cases, during eleven years, assures Dr. Sigmund that secondary symptoms never occur when the chancre has been completely destroyed within the four first days. He is only aware of two doubtful cases in which cauterization on the fifth day even has not prevented accidents. The best caustic is the Vienna, composed of quick lime and two or three parts of caustic potass. Cauterization should also be practised even after the fifth day, for although the chances of preservation from secondary syphilis are diminished, they are not totally abolished; and we prevent the chancre being communicated to other parts of the same patient, or to other individuals. 3. The general treatment consists in the methodical employment of mercury, no other means cure so quickly and so surely. 4. In the exceptional cases in which secondary symptoms occur in spite of general treatment, they are not found in an aggravated form. 5. According to circumstances, the general treatment should be continued for six or twelve weeks. The levity with which the public and the profession at the present time regard venereal symptoms, should be met by the strongest opposition. 6. Clinical observations show that every chancre, well diagnosticated, and not carefully destroyed, leads to secondary symptoms if general treatment has not been instituted. This will be admitted by all who establish a rigorous diagnosis, and look for secondary symptoms soon enough where they are first to be found, viz., in the lymphatic glands. 7. Positive diagnosis is alone attainable by inoculation or the production of secondary symptoms. 8. Secondary symptoms are usually observed about the sixth week after infection, and very rarely later than the twelfth; and we must not always depend upon the patient's assertion, but make ourselves a rigorous search for their early manifestation. If between the sixth and end of the twelfth week no secondary symptom has shown itself, and the local manifestation has disappeared, the patient may be pronounced cured—the few exceptions that occur notwithstanding. 9. The amount of mercury administered varies according to the indications offered by different patients. The dietetic and hygienic management, both during and after taking the mercury, is too much neglected.—*Med. Times and Gazette, from L' Union Médicale,* 1855.

